# Amyand’s hernia associated with mesenteric chylous cyst in infant: a rare case report

**DOI:** 10.1186/s12893-020-00947-w

**Published:** 2020-12-02

**Authors:** Supangat, Muhammad Yuda Nugraha, Meiske Margaretha

**Affiliations:** 1 Division of Pediatric Surgery, dr. Soebandi Regional Hospital-Faculty of Medicine, Jember University, Jember, East Java Indonesia; 2 Faculty of Medicine, Jember University, Jember, East Java Indonesia; 3 Division of Pathological Anatomy, dr. Soebandi Regional Hospital, Jember, East Java Indonesia; 4 Faculty of Medicine, Universitas Jember, Jl Kalimantan No 37, Krajan Timur, Sumber sari Kec Sumber sari, Jember, 68121 East Java Indonesia

**Keywords:** Hernia, Mesenteric cyst, Pediatric, Surgery

## Abstract

**Background:**

Amyand’s hernia is a rare condition approximately 0.4–0.6% of all inguinal hernias. Although rare, the Amyand’s hernia is worthy of discussion since the variable presentation that make clinical challenge to diagnose especially in infant. A mesenteric chylous cyst is rare disease and has not been reported in Amyand’s hernia.

**Case presentation:**

We report an unusual case of Type II Amyand’s hernia with an enlarging chylous mesenteric cyst on the retrocaecal in the anulus into canalis inguinalis. A-2-months old infant presented with enlarging mass in the right scrotal. During laparotomy exploration, we found inguinal sac with intestinal and appendix content in the sac. In the edge site of the sac we found enlarging of mesenteric cyst on the retrocaecal in the anulus into canalis inguinalis. Based on the histopathology examination, the morphological feature is suitable for mesenteric chylous cyst appearance.

**Conclusion:**

Presentation of mesenteric chylous cyst is rare, and there was no report about it in Amyand’s hernia. This unusual presentation should be considered by the surgeon, especially pediatric surgeon, in Amyand’s hernia cases.

## Background

Amyand’s hernia is a rare condition approximately 0.4–0.6% of all inguinal hernias where the vermiform of appendix is found in inguinal hernia sac [1]. An incarcerated or strangulated hernia is commonly presenting in Amyand’s hernia, but it is classified as several types for variable presentation. We report an unusual case of Type II Amyand’s hernia with an enlarging chylous mesenteric cyst on the retrocaecal in the anulus into canalis inguinalis.

## Case presentation

**Fig. 1:**
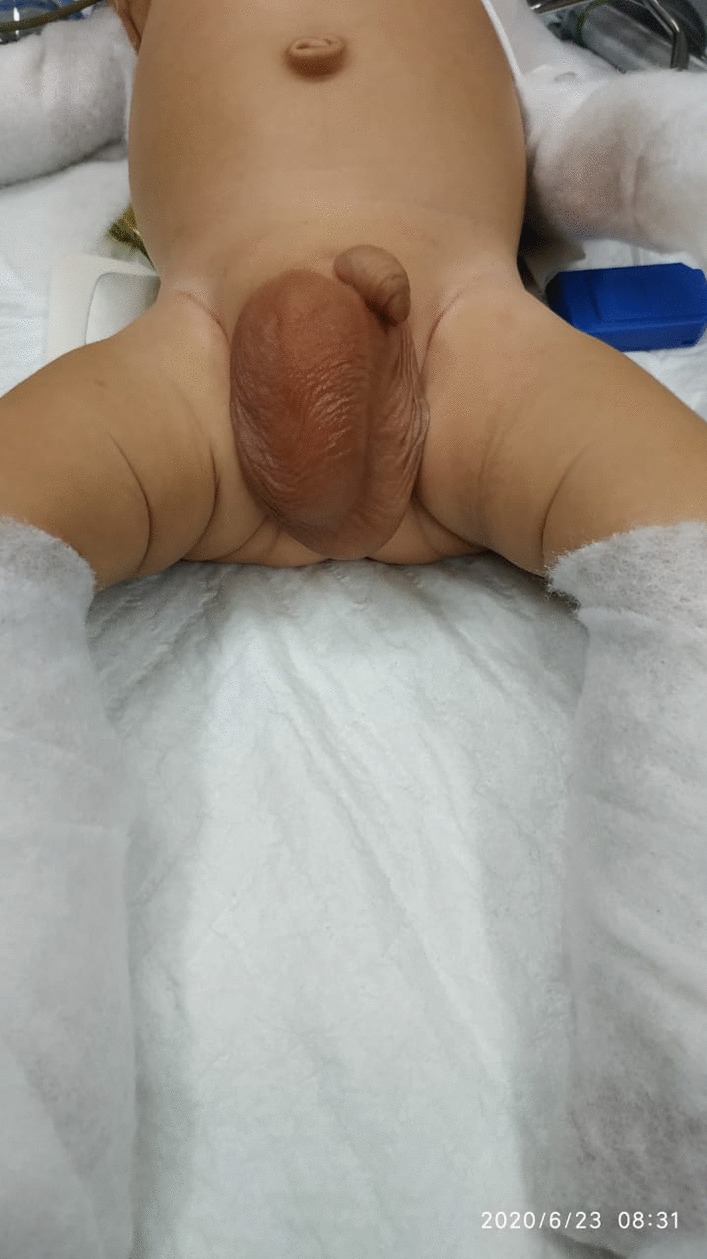
Enlarging mass in the right scrotal

A-2-months old infant presented with enlarging mass in the right scrotal. On physical examination, the enlarging mass was found with diameter 10 cm non-fluctuant, non-erythematous which was fixed and mildly tender to palpation (Fig. 1). Based on hetero anamnesis, the patient vomited with green fluid before presenting into emergency department. The vital sign was steady in normal limit. During abdominal examination, there was decreasing of bowel sounds but there was no abdominal distention sign.

**Fig. 2:**
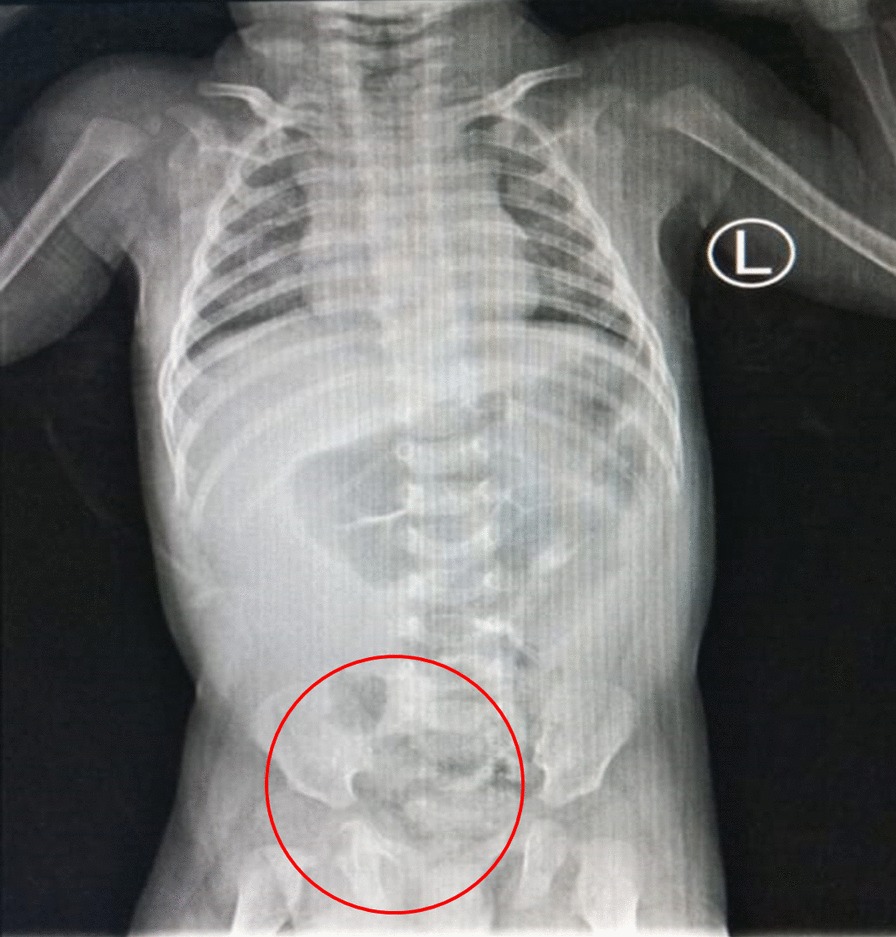
Plain abdominal radiography

In the emergency department the patient underwent to take plain abdominal radiography. There was intestinal like appearance in the scrotal region and some dilated intestinal but no evidence of intestinal obstruction or aeration in the scrotal compartments (Fig. 2). The advance radiological examination cannot be performed since the patient’s condition. All laboratory values were within normal limits. Based on the clinical examination and radiography the patient was diagnosed with Right Scrotum Hernia. After first initial treatment in the emergency department with normal fluid resuscitation, then patient was planned into laparotomy approach to treat the hernia.

**Fig. 3:**
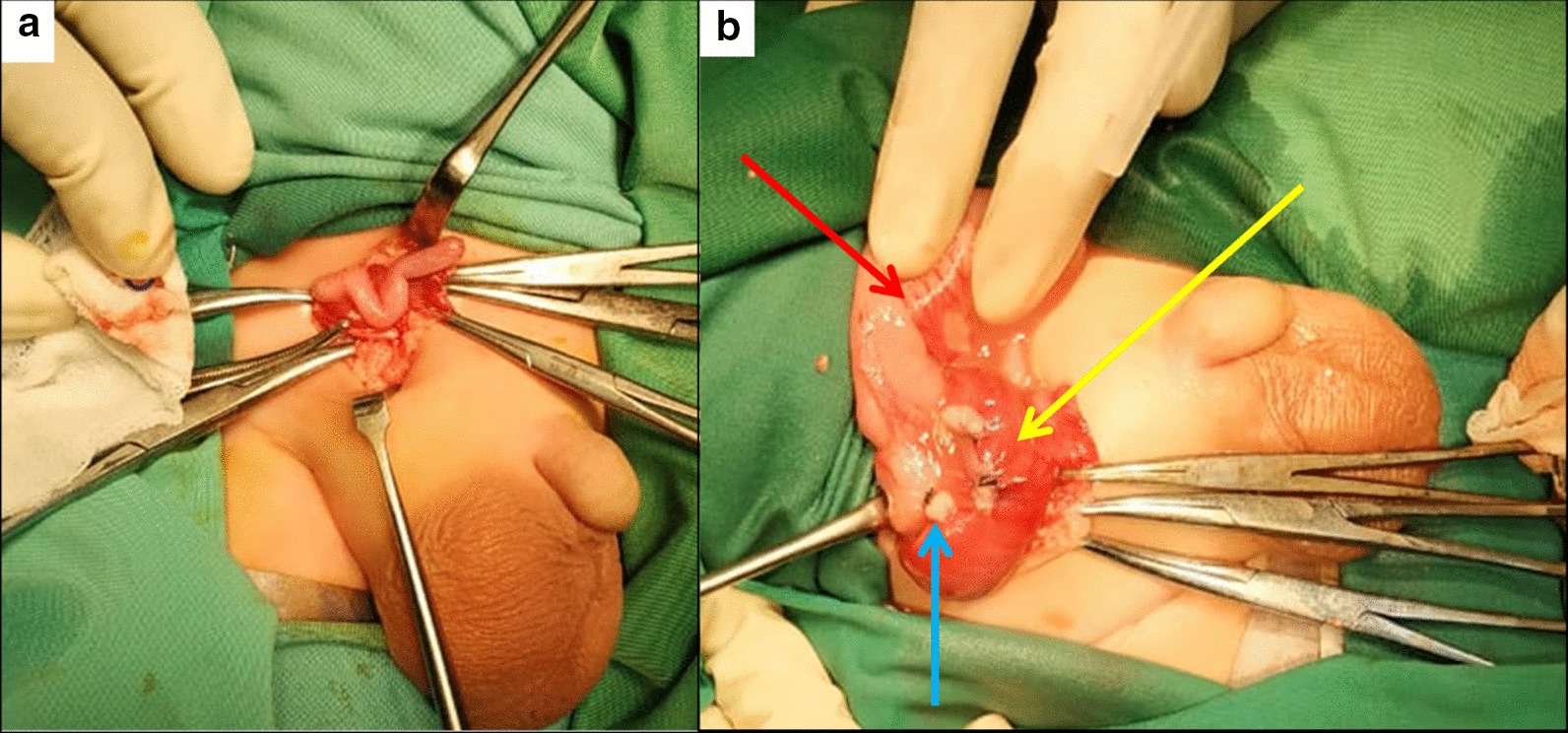
**a** Appendix and intestinal in the inguinal sac, **b** Hyperemia mesenteric cyst (yellow arrow), post appendectomy (blue arrow) and caecum (red arrow) in the inguinal sac

During laparotomy exploration, we found inguinal sac with intestinal and appendix content in the sac (Fig. 3a). The inflamed appendix but there was no perforation sign that indicate the type II of Amyand’s Hernia as intraoperative diagnosis. In the edge site of the sac we found hyperemia mesenteric cyst on the retrocaecal in the canalis inguinalis (Fig. 3b). Appendectomy, cyst resection and hernia closure were done. Then the cyst resection was presented to histopathology examination.

**Fig. 4:**
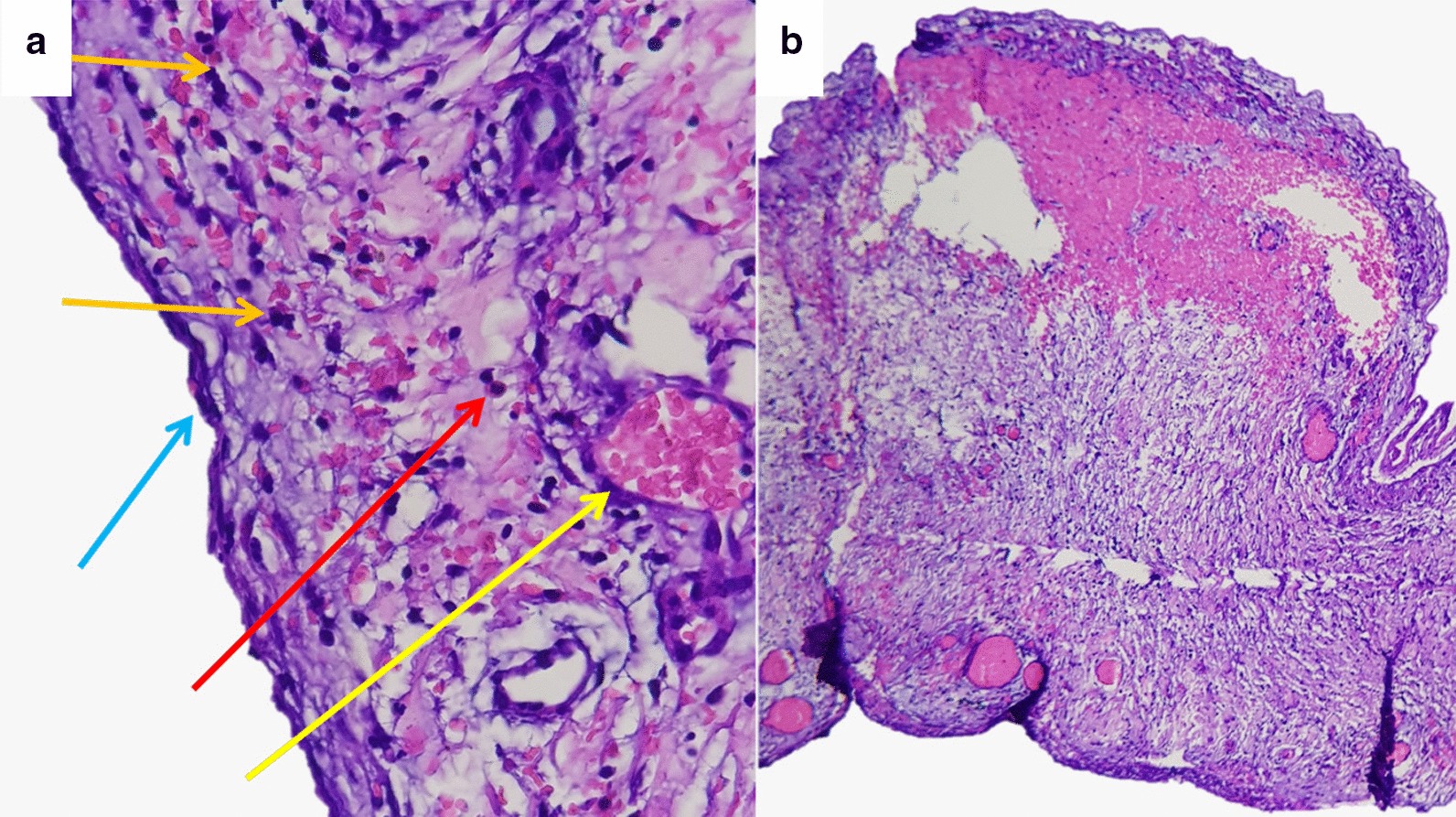
Histopathology examination of cyst resection

Based on the histopathology examination, the cyst resection is form of a swollen connective tissue (red arrow in Fig. 4a) that coated with spindle-shaped cells resembling endothelial cells on the surface (blue arrow in Fig. 4a). Lymphoplasmasitic inflammatory cells appeared in the stroma then also moderate neutrophil PMN type (orange arrow in Fig. 4a) and dilated blood vessels (yellow arrow in Fig. 4a). There was also benign smooth muscle tissue and the large area of bleeding (Fig. 4b). There are no malignant signs on this resection. This morphological feature is suitable for Chylous cyst appearance.

## Discussion and conclusion

In 1735 at the St. hospital George in London, dr. Claudius Amyand succeeded in carrying out the first appendicitis surgery in the world. The patient is an 11-year-old boy with a diagnosis of inguinal hernia combined with acute appendicitis. During the operation, dr. Amyand finds the appendix in the hernia sac [2]. This rare pathology is then given the name "Amyand Hernia" according to the name of the surgeon who first described it and treated it. The incidence of an Amyand’s Hernia is less than 1% of inguinal hernias with the most patients is male. In this case, the patient is infant and male. It is really challenging to diagnose Amyand’s hernia in infant.

Amyand ‘s Hernia commonly located on the right side due to the location of the appendix. Approximately 0.1% of Amyand’s Hernia cases, appendix could be inflamed [3]. Therefore, Losanoff et al. classified Amyand’s hernias into four type. The type I is defined when there is no described appendix inflammation; type II describes acute appendicitis within the hernia sac; type III acute appendicitis complicated with peritonitis; and type IV acute appendicitis is accompanied by other diseases [4]. In this case, we found an acute appendiscitis during intraoperative but there was no sign of peritonitis. Then, the type of Amyand’s Hernia in this case is classified as type II Amyand’s Hernia.

Amyand’s Hernia commonly performs as incarcerated inguinal hernias that make it very difficult to diagnose only by clinical assesment especially in infant. Preoperative diagnosis of Amyand’s Hernia can be established using investigations in the form of ultrasound or CT scan. Mostly cases of Amyand’s Hernia are diagnosed intraoperatively during the operation process [5]. In this case, we diagnosed Amyand’s Hernia intraoperatively. During preoperative diagnosis, we only performed scrotal hernia as a diagnosis based on clinicaly and plain abdominal radiography since abdominal ultrasound or CT Scan cannot be performed.

In Amyand’s Hernia, the appendix can be found together with the right cecum or colon in the hernia sac. In some cases it can also be found a bladder, ovary, fallopian tube, omentum or meckel diverticulum in the hernia sac [6]. In this case we found mesenteric cyst in the hernia sac. After histopathology examination, the type of cyst is classified as mesenteric chylous cyst. The presenting of mesenteric chylous cyst in Amyand’s hernia hasnot been reported before.

Mesenteric cysts is a rare pathologic entity that identified in approximately 1 out of 100,000 adult and 1 in 20,000 paediatric in hospital admissions [7]. A mesenteric chylous cyst is the type of mesentery cyst which may extend from the base of the mesentery into the retroperitoneum [8]. A mesenteric chylous cyst could be totally asymptomatic or present with unspecific abdominal symptoms. It is a rare disease, but surgeons must consider the diagnosis in the presence of a cystic abdominal tumor [9].

Amyand’s hernia has variability in presentation that makes it difficult to diagnose clinically in infant and mostly identified intraoperatively. Presentation of mesenteric chylous cyst is rare, and there was no report about it in Amyand’s hernia. This unusual presentation should be considered by the surgeon, especially pediatric surgeon, in Amyand’s hernia cases.

## Data Availability

Not applicable.

## Abbreviations

CT scan: Computed tomography scan
PMN: Polymorphonuclear
